# Seeing the invisible: Tools to teach and study plant transcriptional responses

**DOI:** 10.1093/plphys/kiae421

**Published:** 2024-08-14

**Authors:** Bikash Baral, Saku Riihelä, Jasmin Kemppinen, Maija Sierla, Mikael Brosché

**Affiliations:** Organismal and Evolutionary Biology Research Programme, Faculty of Biological and Environmental Sciences, and Viikki Plant Science Centre, University of Helsinki, Helsinki FI-00014, Finland; Organismal and Evolutionary Biology Research Programme, Faculty of Biological and Environmental Sciences, and Viikki Plant Science Centre, University of Helsinki, Helsinki FI-00014, Finland; Organismal and Evolutionary Biology Research Programme, Faculty of Biological and Environmental Sciences, and Viikki Plant Science Centre, University of Helsinki, Helsinki FI-00014, Finland; Organismal and Evolutionary Biology Research Programme, Faculty of Biological and Environmental Sciences, and Viikki Plant Science Centre, University of Helsinki, Helsinki FI-00014, Finland; Organismal and Evolutionary Biology Research Programme, Faculty of Biological and Environmental Sciences, and Viikki Plant Science Centre, University of Helsinki, Helsinki FI-00014, Finland

## Abstract

Reporter gene-expressing plants show promoter activity, the stability of transgene expression, and are suitable tools to teach students about plant biotechnology and plant–environment interaction.

Dear Editor,

The term “plant blindness,” coined in 1999, refers to the phenomenon where students prefer to study animals rather than plants (Wandersee and Schussler 1999). In a worst-case scenario, this leads to an inability to realize the importance of plants. To captivate students' attention and stimulate their curiosity about plants, we can develop innovative teaching tools that incorporate living plants, rather than conventional lectures and textbooks. Teaching plant–environment interaction, genetics, and molecular biology can be difficult due to many theoretical concepts including the central dogma and signaling pathways. Comprehension of these theoretical concepts can benefit from a teaching activity where students perform experiments with live plants and the read-out is visible to the naked eye.

Reporter genes/proteins are enzymes that catalyze the formation of colored compounds, bioluminescence, or fluorescence. Examples include GUS (β–glucuronidase) staining (Jefferson 1989), the use of fluorescent proteins including jellyfish green fluorescent protein (Prasher 1995) and light from luciferase (De Ruijter et al. 2003). While being very powerful, these methods have limitations for teaching, including long staining procedures or need of specialized equipment. RUBY is a reporter system that uses three genes, *CYP76AD1* (Cytochrome P450 76AD1), *DODA* (L-DOPA 4,5-dioxygenase), and *glucosyl transferase* to convert the amino acid tyrosine into red/purple betalain (He et al. 2020), the pigment that gives striking color to red beets (Pavlov et al. 2005). Here, we introduce transgenic plants with RUBY expression driven by inducible and constitutive promoters, which provides an easy teaching tool that students can use on a lecture or laboratory course to study the mechanisms behind transcriptional regulation, and plant responses to the environment, pathogens, and hormones.

We constructed promoter-RUBY Arabidopsis (*Arabidopsis thaliana*) lines responsive to a broad range of hormones and treatments, abscisic acid (ABA, *RESPONSIVE TO ABA 18* (*RAB18*; Lehmann et al. 2020), jasmonic acid (*JASMONATE-ZIM-DOMAIN PROTEIN 10* (*JAZ10*; Lehmann et al. 2020), reactive oxygen species (*ZINC FINGER OF ARABIDOPSIS THALIANA12* (*ZAT12*; Lim et al. 2019), and pathogens (*LATE UPREGULATED IN RESPONSE TO HYALOPERONOSPORA PARASITICA* (*LURP1*; Knoth and Eulgem 2008), *WRKY DNA-BINDING PROTEIN 40* (*WRKY40*; Pandey et al. 2010), and *WRKY DNA-BINDING PROTEIN 75* (*WRKY75*; Chen et al. 2021). In addition, we included the constitutive promoters 35S and UBQ10 (Supplementary Material and Methods and Supplementary Table S1). To explore these lines for teaching, we tested a broad range of treatments (Fig. 1 and Supplementary Figs. S1 to S4). In in vitro assays on MS plates, ABA treatment activated *RAB18*-RUBY and methyl jasmonate (MeJA) treatment activated *JAZ10*-RUBY as shown by accumulation of purple pigments (Fig. 1A). ABA also activated *RAB18*-RUBY in soil-grown plants (Fig. 1B). Plants actively monitor the environment, and the RUBY lines can be used as an exploratory tool to test different environmental conditions and treatments, as summarized in Supplementary Table S2. As an example, we used the translational inhibitor cycloheximide and protoplast reagent, containing cellulase and pectolyase. Both treatments activated *WRKY75*-RUBY (Fig. 1C). One of the most studied plant–pathogen systems is the infection of Arabidopsis with *Pseudomonas syringae* pv. tomato strain DC3000 (Pst DC3000; Xin and He 2013). We sprayed RUBY lines with Pst DC3000 and followed RUBY accumulation for 3 days. This led to strong RUBY pigment accumulation in *WRKY75*-RUBY and several other lines (Fig. 1D and Supplementary Fig. S3). While doing experiments with RUBY lines together with students, we have repeatedly been surprised about the activation of RUBY, for example, simply moving plants from controlled growth conditions to classroom was sufficient to activate *RAB18*-RUBY and *WRKY40*-RUBY (Supplementary Fig. S4). This likely resulted from a change in air humidity which can activate the *RAB18* promoter (Merilo et al. 2015). While RUBY is easily observed, the promoter-RUBY lines only accumulate sufficient betalain to be visible by naked eye one day or later after treatment (Supplementary Text and Supplementary Fig. S5).

**Figure 1.**
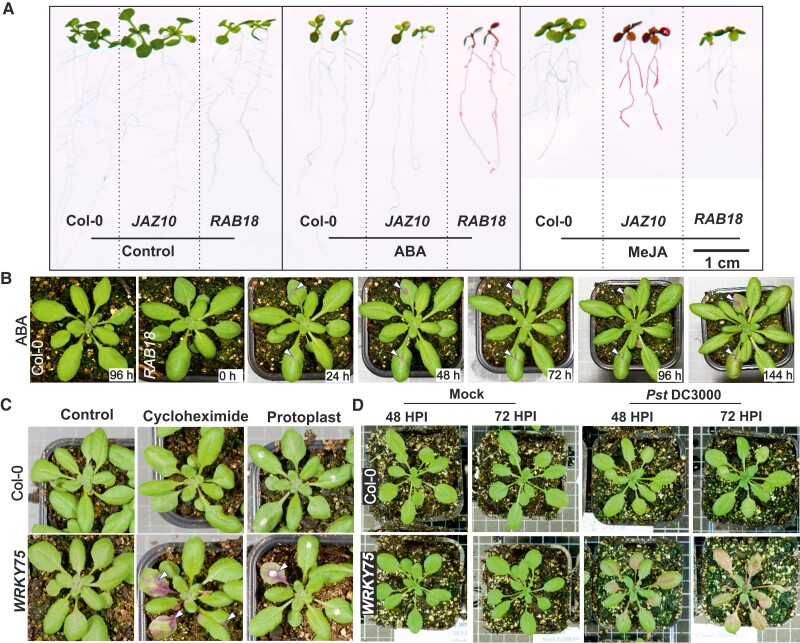
Response of promoter-RUBY lines to different treatments. **A)** In vitro plates supplemented with ABA or MeJA. **B)** ABA treatment of leaves in *RAB18*-RUBY followed for 144 h. **C)** A drop of cycloheximide or protoplast reagent placed on *WRKY75*-RUBY leaves (representative images from Supplementary Fig. S4; i to iii and xxi to xxiii). **D)**  *WRKY75*-RUBY sprayed with Pst DC3000 (representative images from Supplementary Fig. S3; i to iv and xvii to xx). See Supplementary Figs. S1 to S4 for detailed figures and notes.

The 35S and UBQ10 promoters are commonly used in research to generate plants that overexpress a gene of interest. From a research perspective, it has been long recognized that a minimum of two independent transgenic lines should be used for characterization, as the T-DNA insertion can disrupt genes at the insertion site, and local chromatin status can influence the expression of the foreign gene. We used the *35S*-RUBY vector in *Agrobacterium*-mediated gene transfer via floral dipping to Col-0 on lab courses in 2022 to 2024. This is very informative for teaching, as transformed seeds are purple and very easy to spot against the nontransformed seeds. However, the students also observed the plants after transfer to soil, and around 50% or more of the plants displayed gene silencing, where T1 plants transferred to soil initially had purple color but later turned green. We documented this phenomenon in selected T3 lines with 35S promoter, where the silencing often started at one leaf and eventually spread throughout the entire plant (Fig. 2 and Supplementary Figs. S6 and S7). UBQ10 was introduced as a better promoter for transient and stable expression of native or foreign genes in tobacco and Arabidopsis (Grefen et al. 2010). However, the *UBQ10*-RUBY lines showed clear age regulation, where older plants were more purple (Fig. 2).

**Figure 2.**
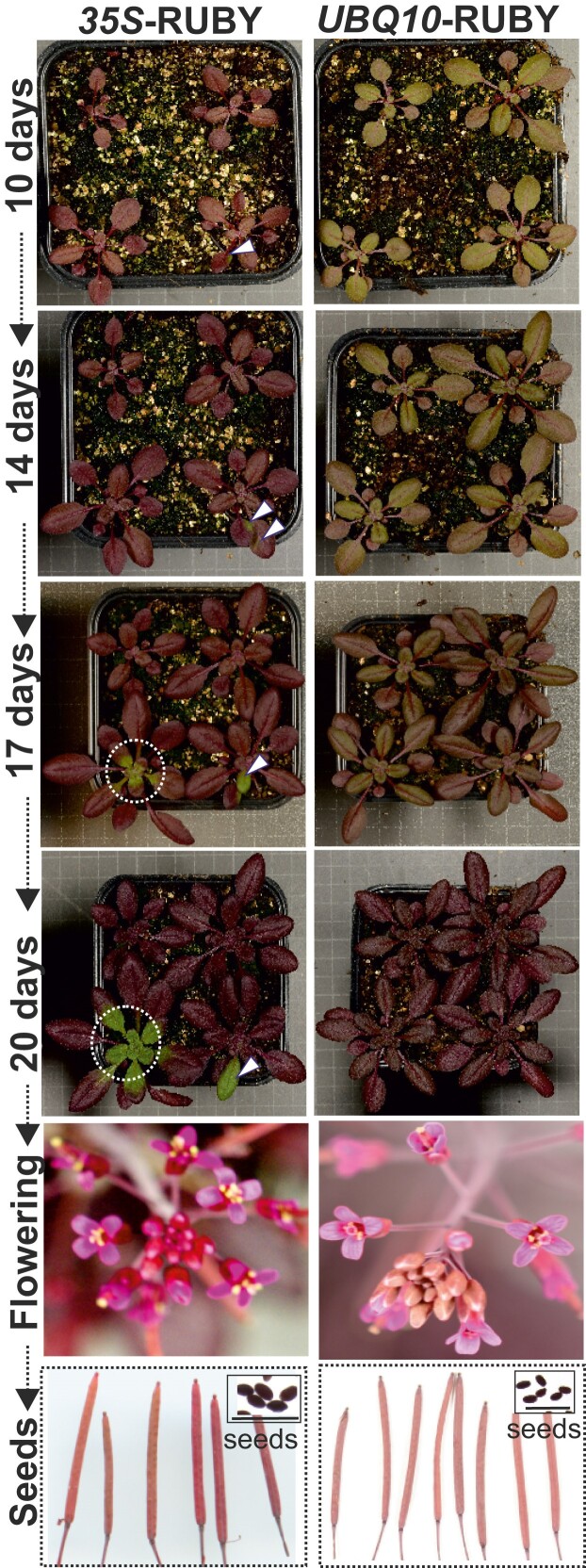
Constitutive promoters *35S*-RUBY and *UBQ10*-RUBY. A high proportion of *35S*-RUBY plants show silencing (white arrows and circles). Representative images from Supplementary Fig. S6 were taken. *35S*-RUBY line A2-7-1 line (panels i, ix, xvii, and xxv) and *UBQ10*-RUBY line E11-1 (panels viii, xvi, xxiv, and xxxii). Flowers and siliques images taken from Supplementary Fig. S6, (for flowers: panels ii and xvii, and for siliques: panels iii and xviii). For more lines and notes, see Supplementary Figs. S6 to S7. Scale-bars: 50 µm.

To engage students in the scientific process, we suggest that teachers together with students can design experiments with the RUBY lines to make new discoveries. The promoters used here should respond to a broad range of stimuli as judged by gene expression data from the Genevestigator database (Supplementary Fig. S8). Thus, while we propose that simple experiments can include ABA treatments, and chemicals to activate signaling, we encourage experiments that include treatments suggested by students to explore how plants respond to the environment. A minimum experiment would include the best responding lines *RAB18*-RUBY and *WRKY75*-RUBY. The *35S* and *UBQ10*-lines can be included if the course includes topics related to plant biotechnology or gene silencing. Our experience is that many students find the 35S half-green, half-purple plants interesting and these lines could be used as an easy way to start discussion with students about plant transformation and gene silencing.

## Supplementary Material

kiae421_Supplementary_Data

## Data Availability

All transgenic lines generated in this study are deposited to the Nottingham Arabidopsis Stock Centre (NASC), UK with the accession codes (N2112038–N2112054), and can also be made available from the corresponding author M.B. (mikael.brosche@helsinki.fi) upon request.

## Supplementary data

The following materials are available in the online version of this article.

**Supplementary Table S1.** Oligonucleotide sequences and primers used in the study.

**Supplementary Table S2.** Percentage of plants from Supplementary Figs. S1 to S4 showing RUBY at 72 h after treatment.

**Supplementary Figure S1.**  *In vitro* assays for promoter-RUBY lines in response to abscisic acid (ABA) and methyl jasmonate (MeJA).

**Supplementary Figure S2.** Response of soil-grown Col-0 and *RAB18*-RUBY to ABA (50 µM).

**Supplementary Figure S3.** Response of promoter-RUBY lines to pathogen infection.

**Supplementary Figure S4.** Response of promoter-RUBY lines to chemicals and humidity shift.

**Supplementary Figure S5.** How to see the difference between anthocyanin versus RUBY.

**Supplementary Figure S6.** RUBY expression driven by constitutive promoters (*35S* and *UBQ10*) in leaves and flowers.

**Supplementary Figure S7.** Constitutive promoters *35S* and *UBQ10*-RUBY in vitro.

**Supplementary Figure S8.** Gene (promoter) expression in different treatments retrieved from Genevestigator.

Supplementary Materials and Methods.

**Supplementary Text.** Limitations of the RUBY reporter system, and reproducibility of the promoter-RUBY lines.

## Dive Curated Terms

The following phenotypic, genotypic, and functional terms are of significance to the work described in this paper:

RAB18 Gramene: AT5G66400
RAB18 Araport: AT5G66400

LURP1 Gramene: AT2G14560
LURP1 Araport: AT2G14560

WRKY40 Gramene: AT1G80840
WRKY40 Araport: AT1G80840

WRKY75 Gramene: AT5G13080
WRKY75 Araport: AT5G13080

JAZ10 Gramene: AT5G13220
JAZ10 Araport: AT5G13220

ZAT12 Gramene: AT5G59820
ZAT12 Araport: AT5G59820
