# Supplementary material for: Seeing the invisible: Tools to teach and study plant transcriptional responses
Source: Plant Physiol. 2024 Aug 14;196(3):1729–32. doi: 10.1093/plphys/kiae421 (PMC11531832; doi:10.1093/plphys/kiae421)
Supplement: kiae421_Supplementary_Data [file kiae421_supplementary_data.zip › PP2024RA00643R1_Supplemental_Material 1.docx]

***Supplementary DATA***

**Seeing the invisible: tools to teach and study plant transcriptional responses**

Bikash Baral, Saku Riihelä, Jasmin Kemppinen, Maija Sierla and Mikael Brosché*

Organismal and Evolutionary Biology Research Programme, Faculty of Biological and Environmental Sciences, and Viikki Plant Science Centre, University of Helsinki, FI-00014 Helsinki, Finland

*Author for correspondence: Mikael Brosché (Email: mikael.brosche@helsinki.fi)

**SUPPLEMENTARY Materials and Methods**

*Plant materials and chemicals*

Seeds for Arabidopsis (*Arabidopsis thaliana*) Columbia-0 (Col-0) were obtained from the Nottingham Arabidopsis Stock Centre, UK. All chemicals used in the current study were purchased from Sigma Aldrich, unless otherwise stated.

*Growth conditions of soil-grown plants*

Seeds were cold treated at +4°C for three days and then grown in 1:1 peat-vermiculite under 280 µmol m^2^/s white light irradiance, 12 h: 12 h light-dark –cycle, 23°C/19°C (day/night) temperature and 70%/90% relative humidity in growth rooms or Weiss chambers. Three-week-old plants were used for various treatments.

*35S* and *UBQ10*-RUBY lines were photographed at day 10 and every 3 days until day 20, capturing phenotypic variations among the different plants. To initiate flowering, 20-day-old plants were transferred to greenhouse with 18 h : 6 h light-dark –cycle, but as the greenhouse compartment did not have curtains, the length of light period was longer in summer. Images of the inflorescence were captured upon the emergence and flowering of the main apical inflorescence. Siliques were photographed before seed harvesting.

*Construction of promoter-RUBY lines*

The RUBY unit including HSP18.2 terminator from Arabidopsis was amplified with Phusion polymerase (ThermoFisher Scientific) from *35S*-RUBY (Addgene plasmid 160908; (He et al., 2020)) using primers (Suppl. Table S1) that contain *Bsa*I restriction enzyme sites to allow GreenGate cloning (Lampropoulos et al., 2013). PCR products were purified, digested with *Bsa*I and inserted to the “CDS” pGGC000 vector of the GreenGate system. Promoters were amplified from Arabidopsis Col-0 DNA using primers (Suppl. Table S1), that contain *Bsa*I and cloned to the promoter pGGA000 vector in the GreenGate system. The resulting entry vectors were sequenced (StarSEQ, Germany). The 35S and UBQ10 promoters come from the GreenGate vector set (plasmids pGGA006 and pGGA004). Destination vectors for plant transformation were assembled in the pGGZ003 backbone, using the promoter vector in position A, dummy in position B (pGGB003), RUBY in position C, dummy in position D (pGGD002), HSP18.2 terminator in position E (pGGEX001) and BASTA (active component phosphinothricin) resistance in position F (pGGF001). Cloning to destination vector was done with New England Biolabs Golden Gate Assembly Kit (*Bsa*I-HF®v2). Correct assembly into destination vectors was verified with PCR and final destination vectors were transformed to *Agrobacterium tumefaciens* GV3101(pMP90). Arabidopsis Col-0 was transformed with floral dip (Clough and Bent, 1998). T1 plants were selected for BASTA resistance and presence of RUBY with PCR (oligos in Supplemental Table S1). In T2 generation, plants were selected for BASTA segregation to pick lines with a single T-DNA insert, and for expression of RUBY using various stress and hormone treatments (including treatments with the air pollutant ozone, salicylic acid, methyl jasmonate, pathogens and senescence). Only lines that showed RUBY colour were continued to identify homozygous T3 seeds which were used for further experiments.

*In vitro hormone treatments*

For *in vitro* assays, plants were grown for 1 week on ½ MS-2 g/L (w/v) Gellan media (pH 5.7) in Panasonic growth chamber 16 h : 8 h light-dark cycle. One-week-old seedlings were transferred to control plates or treatment plates supplemented with 50 µM ABA or 50 µM MeJA and observed for further ten days. Plants were scanned using SilverFast software and EPSON scanner (Epson Perfection V750 Pro).

*Chemical and hormone treatments*

Water (mock), ABA (50 µM) and NaCl (200 mM) treatment on soil grown plants was performed on 19-day-old plants by placing two 20 µL drops on the adaxial side. Too high NaCl concentration (>500 mM), should be avoided as it leads to plant damage. Plants were photographed daily up to 144 h of treatment.

Water (mock), cycloheximide (50 µM) or protoplasting reagent (500 mM mannitol, 2% sucrose, 0.5% MES pH 5.7, 0.5% cellulase, 0.2% pectolyase) treatment on soil grown plants was performed on three-week-old plants by placing two or three 10 µL drops on the adaxial side. Plants were photographed at 48 h and 72 h of treatment. Three-week-old plants were also transferred from growth chamber (70% humidity) to ambient conditions (office ≈25% humidity, lights on during working hours), and photographed 1 week after transfer.

*Pathogen infection*

To investigate RUBY lines in response to *Pseudomonas syringae* pv. tomato DC3000 infection, 3.5-week-old plants were infected by spray inoculation and the visual symptoms were inspected after 48- and 72-hours post-infection (HPI). A bacterial culture was prepared from one colony in 5 mL liquid LB (Luria-Bertani broth), which was left shaking overnight in 180 rpm at 28^○^C. Subsequently, a 1:50 dilution of the overnight culture was prepared in liquid LB and left shaking at 28^○^C for 12 h.

On the day of infection, plants were covered with a transparent lid for 2 h to enhance humidity and promote stomatal opening. Bacterial pellets were collected by centrifuging at 4000 rpm for two minutes and resuspended in 10 mM MgCl_2_. Centrifugation and resuspension steps were repeated twice. The final spraying solution was adjusted to a final OD_600_ of 0.2 in 10 mM MgCl_2_, and to reduce surface tension, Silwet was added to the spray solution to achieve a final concentration of 0.02%. Additionally, a control solution consisting of 10 mM MgCl_2_ with 0.02% Silwet was prepared.

The adaxial surfaces of plants were thoroughly saturated with an airbrush spray at mid-day either with Pst DC3000 or control solution with separate designated tanks and nozzles, and the plants were covered with a transparent lid to increase humidity, essential for disease development.

# *Confirmation for the betalains from 35S-RUBY transgenic lines of A. thaliana*

*A. thaliana* plants when stressed may generate anthocyanins which are also purple in color similar to betalains. Thus, to ascertain the pigment produced by the transgenic RUBY lines are betalains from the RUBY pathway and not stress-induced anthocyanins, extraction of the pigments was done from three weeks old leaves of 35S purple RUBY lines. Arabidopsis Col-0 of the similar age acted as a negative control. For the extraction of the pigments, leaves were ground in 75% (v/v) ethanol (pH: 5.2) using glass beads and the extraction was further continued for 1 hour with continuous stirring. Following the extraction, the mixture was centrifuged (12,000 × *g*; 20 min at room temperature) and the obtained supernatant was analyzed using Nanodrop spectrophotometer. The absorption spectrum of the betalain pigment was plotted using OriginPro 2024.

**supplementary text**

Although RUBY is easily observed, it also has three limitations that should be considered while planning experiments for students: (1) For the inducible promoters used in this study, and in all treatments we have tested, there has never been accumulation of sufficient RUBY color on the same day of the treatment to be observed by a naked eye. This means that use of promoter-RUBY lines is at a minimum a two-day experiment, where treatments are done on the first day, and observation of RUBY on the second day. As an additional example, see Supplemental Fig. S5 for treatments with ozone and syringe infiltration of boiled *E. coli*, which were used in the initial screening to identify RUBY lines that respond well to treatments. (2) As the promoter-RUBY lines are generated through *Agrobacterium*-mediated transformation and T-DNA transfer they are GMO, and compliance with local and national GMO jurisdiction will be required for their use. (3) Arabidopsis accumulate anthocyanins in response to stress and during ageing (Supplemental Fig. S5). The RUBY color betalain, should not be confused with anthocyanin. We suggest two ways to control for this: (A) Use Col-0 side by side with RUBY lines. If purple color is seen only in RUBY lines, and not in Col-0, the color is likely to be betalain. (B) The UV/Vis-spectrum is different for betalain (UV/Vis_max_ at 534 nm) (Chaux-Gutiérrez et al., 2021) and anthocyanin (UV/Vis_max_ at 512 nm) (Young and Abdel-Aal, 2009) which can easily be determined with a leaf extract and spectrophotometer (Supplemental Fig. S5).

We assessed the reproducibility of the inducible promoter-RUBY lines in different treatments by counting the number of plants and calculated the percentage of plants that show RUBY accumulation in response to treatments (Supplemental Table S2) at 72 hours post treatment. Most transgenic lines generated showed high reproducibility; when treatment-induced RUBY accumulation was observed in a given line, it was most often observed in all (100%) of the individuals. However, some treatments were less efficient, for example NaCl. *LURP1*-RUBY showed no induction from the treatments used in Supplemental Figs. 1-4, but in other treatments using different types of pathogen-derived elicitors we have observed RUBY also in these lines. WRKY75 is a regulator of senescence, and expression of *WRKY75* is highly induced during senescence (Guo et al., 2017). Accordingly, *WRKY75*-RUBY lines accumulate strong red-purple colors in older leaves (Supplemental Fig. S5). This can occasionally also be observed in the oldest leaf also in younger plants. The roots of *ZAT12*-RUBY and *WRKY75*-RUBY lines displayed RUBY under *in vitro* control conditions and in soil grown plants. Thus, these lines cannot be used for performing treatment-induced root assays (either in MS-plates or in soil).

| Supplementary Table S1. Oligonucleotide sequences and primers used in the study | | | | |
| --- | --- | --- | --- | --- |
| **Locus name /organism** | **Alias** | **Primers** | **Sequence (5’-3’)** | **Purpose** |
|  | RUBY for greengate | Forward | AACAGGTCTCAGGCTatggatcatgcgaccctcgcc | Cloning to pGGC000 |
|  | RUBY rev greengate | Reverse | AACAGGTCTCACTGAtcttatctttaatcatattcc |  |
| AT5G13220 | *JAZ10* | Forward | AACAGGTCTCAACCTcaactataggttcaattata | Cloning to pGGA000 |
| AT5G13220 | *JAZ10* | Reverse | AACAGGTCTCATGTTcttctttgatcttattagaaa |  |
| AT2G14560 | *LURP1* | Forward | AACAGGTCTCAACCTGTCAAATTTTATTTTTTGCATA | Cloning to pGGA000 |
| AT2G14560 | *LURP1* | Reverse | AACAGGTCTCATGTTactttgttttcccctccaaaa |  |
| AT5G13080 | *WRKY75* | Forward | AACAGGTCTCAACCTttaggaacaaagtttgtggatt | Cloning to pGGA000 |
| AT5G13080 | *WRKY75* | Reverse | AACAGGTCTCATGTTattcctaattatttgtggaatc |  |
| AT5G59820 | *ZAT12* | Forward | AACAGGTCTCAACCTgtaatggttttgaaatcatata | Cloning to pGGA000 |
| AT5G59820 | *ZAT12* | Reverse | AACAGGTCTCATGTTttttcttctgatgatgatgatta |  |
| AT1G80840 | *WRKY40* | Forward | AACAGGTCTCAACCTtgcatgtgttattagttatgga | Cloning to pGGA000 |
| AT1G80840 | *WRKY40* | Reverse | AACAGGTCTCATGTTgtaaatatatgtaggatgaat |  |
| AT5G66400 | *RAB18* | Forward | AACAGGTCTCAACCTtctctttaccaaccaactaatc | Cloning to pGGA000 |
| AT5G66400 | *RAB18* | Reverse | AACAGGTCTCATGTTgttcttcttgtcttaagcaaa |  |
|  | Internal RUBY | Forward | gtccgacatcatcaacctgc | PCR verification of vectors and plants |
|  | Internal RUBY | Reverse | atcatcttcataggcccggg |  |

**Supplementary Table S2**

**
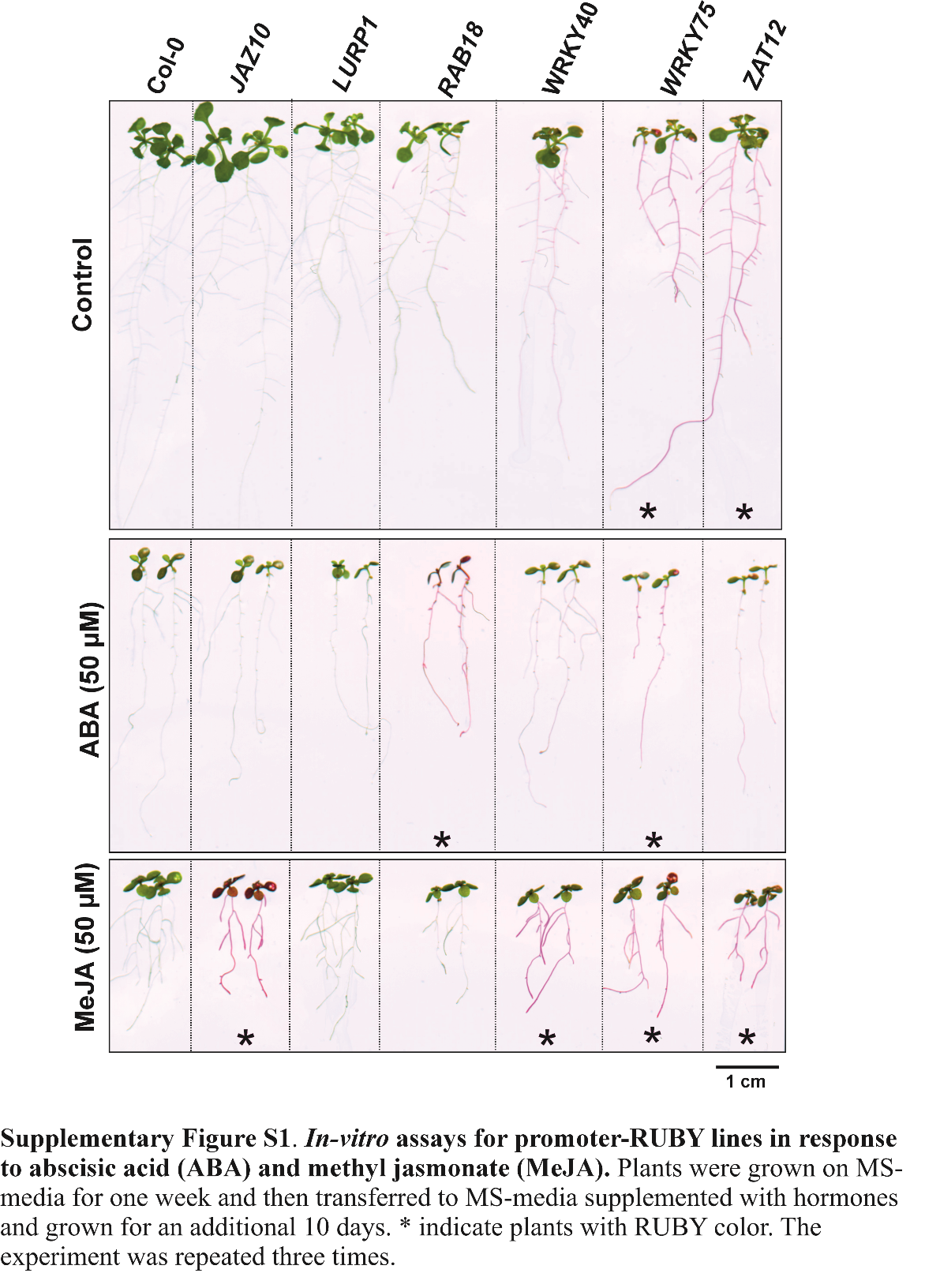
**

**
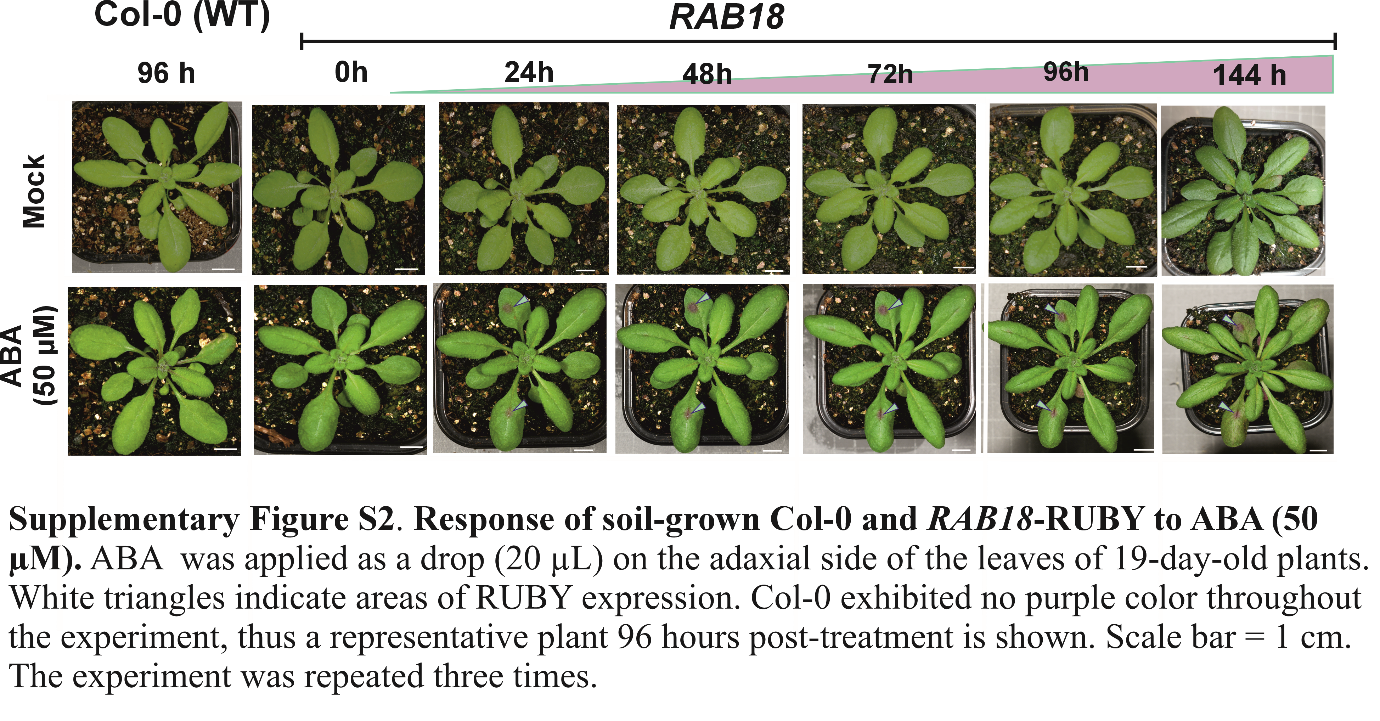
**

**
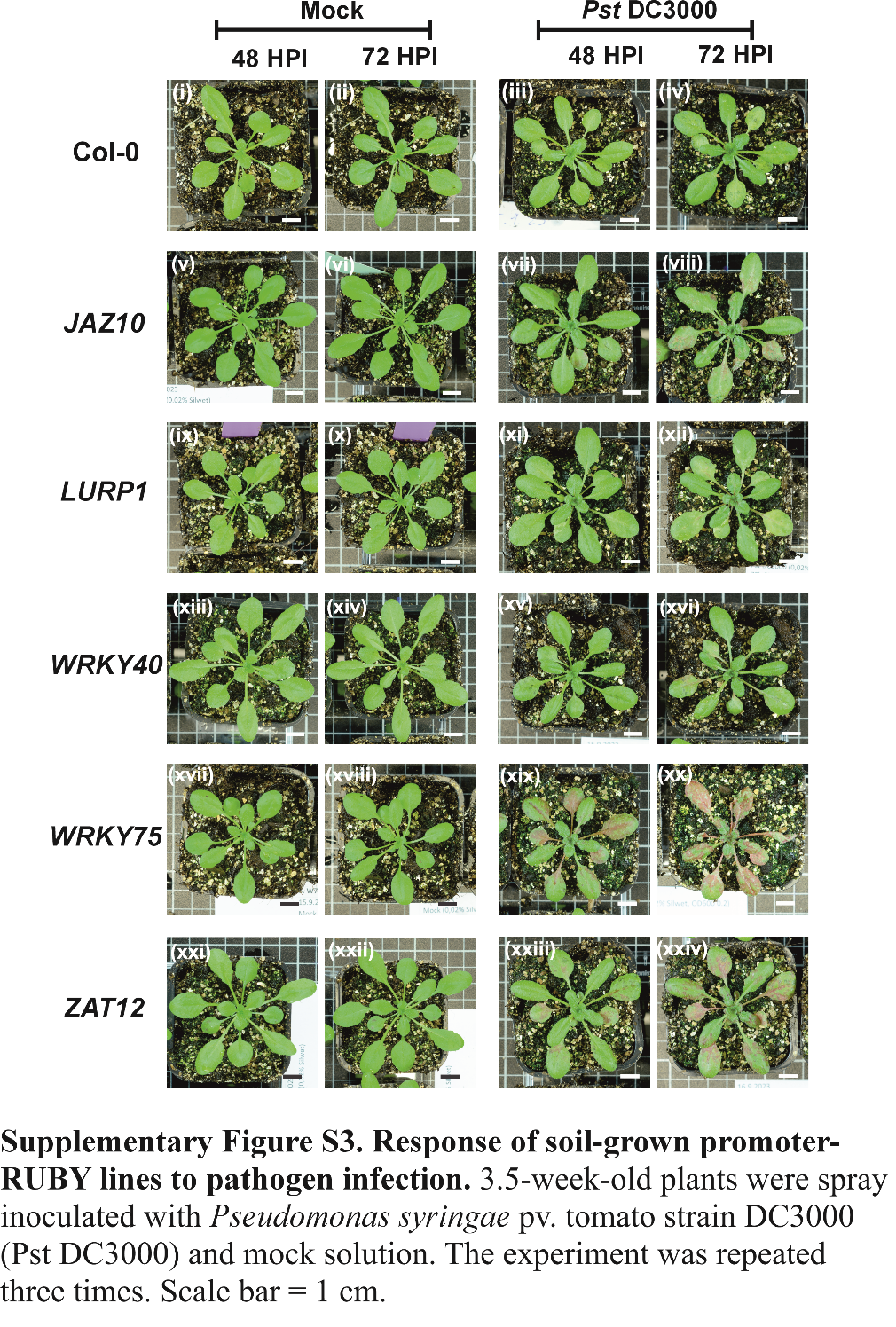
**

**
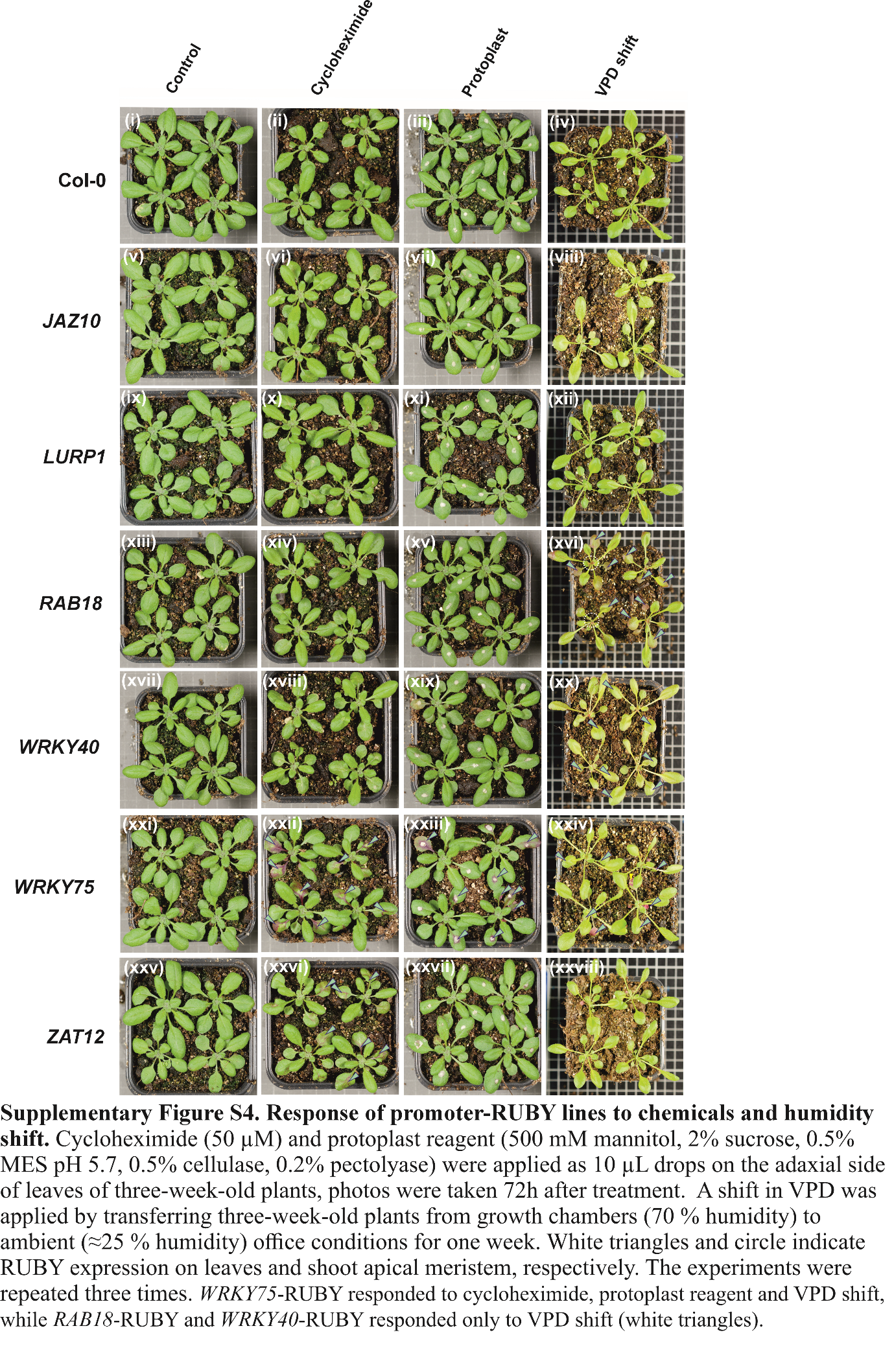
**

**
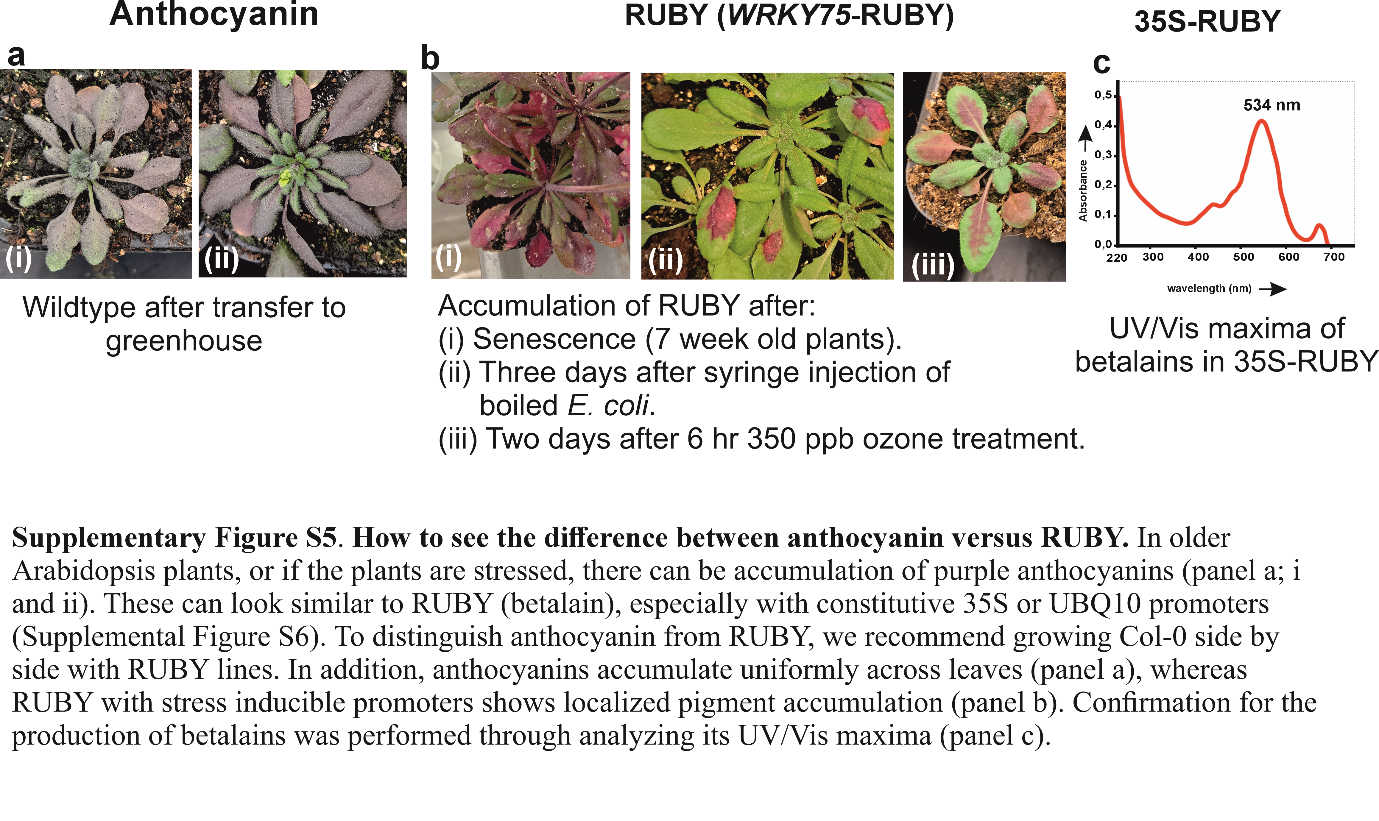
**

**
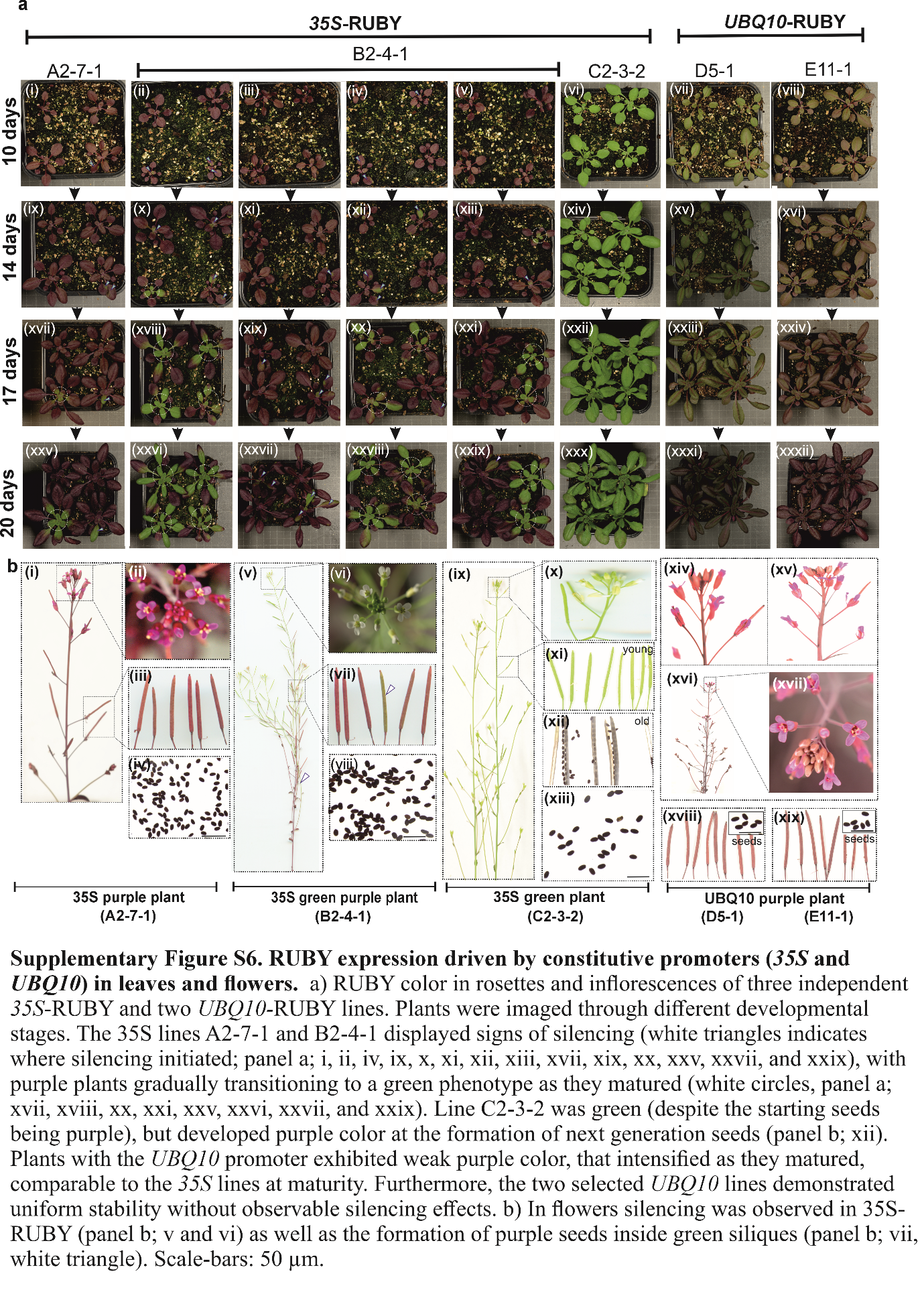
**

**
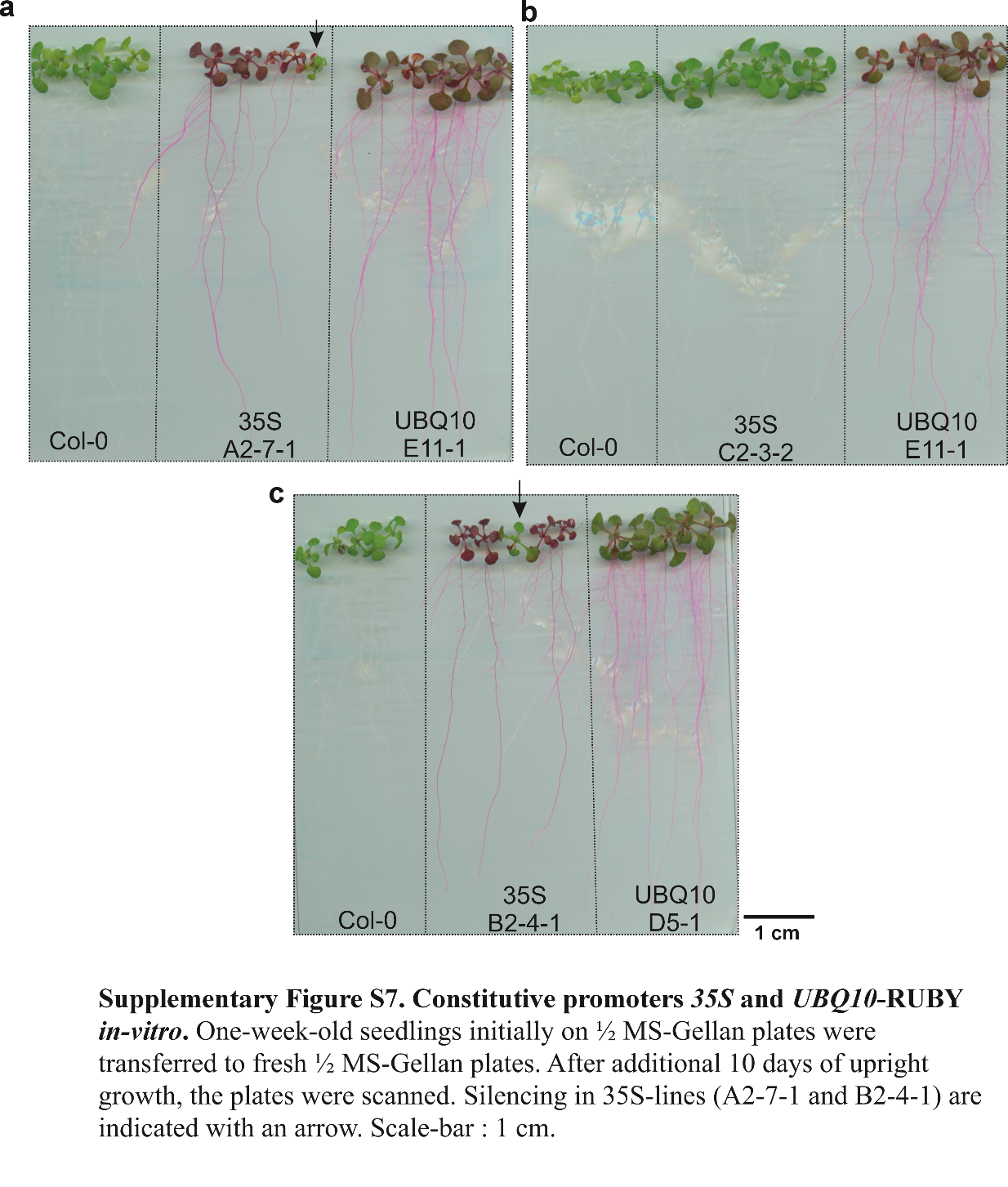
**

**
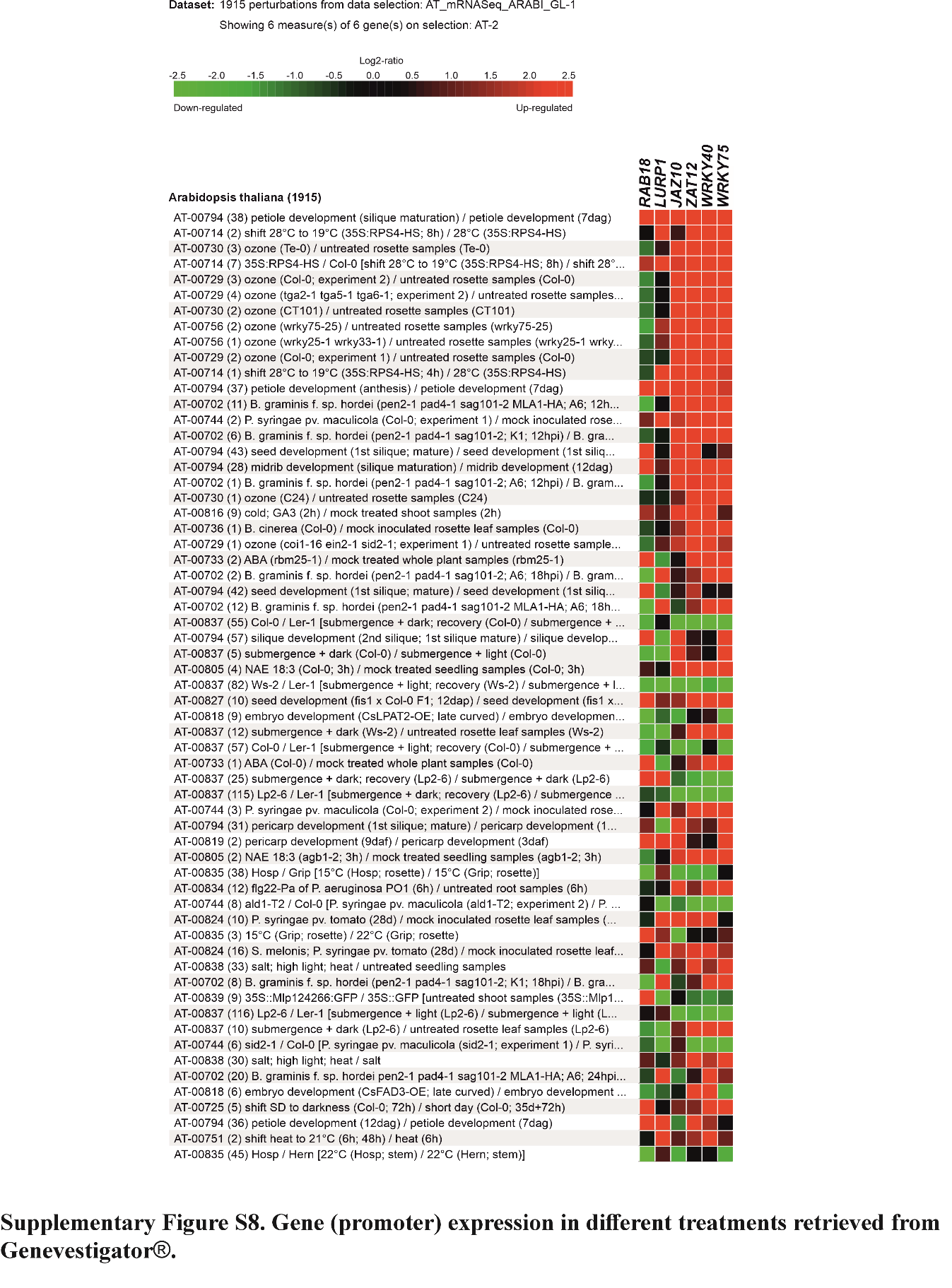
**

**references**

**Chaux-Gutiérrez AM, Pérez-Monterroza EJ, Granda-Restrepo DM, Mauro MA** (2021) Effect of temperature and relative humidity on the stability of betalains encapsulated in cryogels from protein and polysaccharide. J Food Sci Technol **58**: 2007–2018

**Clough SJ, Bent AF** (1998) Floral dip: a simplified method for *Agrobacterium*-mediated transformation of *Arabidopsis thaliana*. The Plant Journal **16**: 735–743

**Guo P, Li Z, Huang P, Li B, Fang S, Chu J, Guo H** (2017) A Tripartite Amplification Loop Involving the Transcription Factor WRKY75, Salicylic Acid, and Reactive Oxygen Species Accelerates Leaf Senescence. The Plant Cell **29**: 2854–2870

**He Y, Zhang T, Sun H, Zhan H, Zhao Y** (2020) A reporter for noninvasively monitoring gene expression and plant transformation. Horticulture Research 2020 7:1 **7**: 1–6

**Lampropoulos A, Sutikovic Z, Wenzl C, Maegele I, Lohmann JU, Forner J** (2013) GreenGate - A novel, versatile, and efficient cloning system for plant transgenesis. PLoS One **8**: e83043

**Young JC, Abdel-Aal ESM** (2009) ANTHOCYANINS. HEALTHGRAIN Methods: Analysis of Bioactive Components in Small Grain Cereals 141–165
