# Supplementary material for: Seeing the invisible: Tools to teach and study plant transcriptional responses
Source: Plant Physiol. 2024 Aug 14;196(3):1729–32. doi: 10.1093/plphys/kiae421 (PMC11531832; doi:10.1093/plphys/kiae421)
Supplement: kiae421_Supplementary_Data [file pp.00643.2024-s05.pdf › pp.00643.2024-s05.pdf]

Plant Physiology®

Seeing the invisible - new tools to teach and study plant transcriptional responses

Bikash Baral, Saku Riihelä, Jasmin Kemppinen, Maija Sierla, and Mikael Brosche

Review Timeline:

Submission Date:	26-Apr-2024
Editorial Decision:	22-May-2024
Revision Received:	21-Jun-2024
Accepted:	08-Jul-2024

22-May-2024

Dr. Mikael Brosche
Helsingin yliopisto
Helsinki 00014
Finland

RE: Seeing the invisible - new tools to teach and study plant transcriptional responses

Dear Dr. Brosche:

We have received reviews of your manuscript, which you may view at the end of this letter. On the basis of the advice received and my own evaluation, I believe that, pending revision, this article may ultimately be acceptable for publication in Plant Physiology.

The reviewers have several constructive suggestions. Reviewer 2 suggested to convert the manuscript into a Letter. I agree with the recommendation because your work did not provide new mechanistic insights, but the materials will be very useful for teaching.

When you are ready to submit your revision, click the below link and find the red arrow under your Author Tasks heading for this revision submission.

If you want to visit this version of the manuscript, you may click the link below and then click on the folder that reads "Post Decision Manuscripts".

Link Not Available

When uploading the revised version of this article, please be sure to include as complete an answer as possible to the referees' criticisms during the first round of reviews in the "response to reviewers" section.

Plant Physiology considers a revision for up to 60 days after a manuscript has been provisionally accepted; limited extension of this timeframe is at the discretion of the Monitoring Editor. A reminder notice will be sent to you 2 weeks before the deadline, at which time you may choose to contact the editorial office to request an extension; if so, please provide a justification for your request. After the expiration of this deadline, the article would need to be submitted as a resubmission and would be handled as such.

Thank you very much for giving us an opportunity to review this work. I look forward to receiving the next version.

Sincerely,

Plant Physiology

Want to add this revision deadline to your calendar? Click below!

-- Reviewer comments:
Reviewer #1 (Comments for the Author):

In this study, different promoters were selected to transcribe RUBY, such as RAB18, JAZ10, ZAT11 (ROS reporter sensor), promoters WRKY40, WRKY75 (involved in various environmental stress regulation processes), and 35S and UBQ10 (constitutive promoters). These vectors were transferred into Arabidopsis Thaliana by flower infection, and two T3 lines were selected to evaluate the effects of ABA, MeJA, biological stress, and chemical stress on the expression of different Promoter-RUBY transgenic events. During the construction of 35S-RUBY stable coloring Arabidopsis Lines, gene silencing occurred in many lines, which may be caused by epigenetic modification. In addition, 35S-RUBY plants showed hyponastic growth. These

were worthy for teaching discussion. I have the following concerns that need to be addressed by the authors.

Major concerns
1. It is better to use UBQ10-RUBY stable line as positive control in some treatment or stress experiment.

2. Please use statistical numbers to show the significance of the influence of the treatment or stress on promoter transcription? Such as the percentage of RUBY color plants/total treated plants.

3. The production of genetically modified materials is a general situation for all types of genetic transformation materials, and should not be a special problem for RUBY.

4. Some other literature has shown that strongly expressed RUBY can affect plant growth. In this experiment, multiple promoters were used to transcribe RUBY, but there was no relevant explanation. Please show and explain the effect of strongly expressed RUBY on plant growth rate and plant size.

5. Figure 5 panel b xii is red seeds produced by green plants? Control photos of plants, inflorescences and seeds identical to T0 and T1 of WT are required.

6. In discussion, the author mentioned the UV/Vis-spectra is different for betalain (UV/Vismax at 534 nm) (Chaux-Gutiérrez et al., 2021) and anthocyanin (UV/Vismax at 512 nm) (Young and Abdel-Aal, 2009). Please show some results about this from your experimental plants.

Minor concerns
1. In Figure 4, the plant styles selected in the photo should be consistent, single plant, two plants, or multiple plants.

Reviewer #2 (Comments for the Author):

RUBY as a reporting system to develop plant materials for teaching purposes is an excellent concept. However, as mentioned in the discussion section, the use of promoter-driven expression of marker genes through different signaling pathways presents a challenge due to longer response times. This is the disadvantage of transcriptional regulatory systems. The manuscript provides only a brief analysis of the phenotype and application specificity of the reporting system, and its scientific and theoretical innovation significance does not meet the scope for a Research Article in Plant Physiology. I recommend considering a submission to Plant Physiology Letters instead. Several issues need to be addressed:

Lines 79-81 are duplicated.
The photo in Figure 2 is too dark; increasing the overall brightness is recommended.
As shown in Figure 3 (XX), the plant has bolted, making it unsuitable for bacterial infection experiments.
To detect reporter gene expression more rapidly, the authors might consider increasing the concentration of hormones or stress treatments, allowing students to observe the phenotype within class hours.

Dear

Plant Physiology

We have submitted the revised version of our manuscript 'Seeing the invisible – new tools to teach and study plant transcriptional responses' by Baral et al. We would like to express our sincere gratitude to the editor and reviewers for their thoughtful comments and suggested edits, which were helpful in preparing the revised version of our manuscript. We have carefully edited the manuscript according to your comments.

Below is a more detailed response to the specific reviewer/editor comments. Our response is indicated in text with grey background. As the manuscript was suggested to be appropriate for the Plant Physiology Letter format, we have modified it accordingly to fit this shorter format.

With best wishes,

Mikael Brosché on behalf of co-authors

Editor comment:
We have received reviews of your manuscript, which you may view at the end of this letter. On the basis of the advice received and my own evaluation, I believe that, pending revision, this article may ultimately be acceptable for publication in Plant Physiology.

The reviewers have several constructive suggestions. Reviewer 2 suggested to convert the manuscript into a Letter. I agree with the recommendation because your work did not provide new mechanistic insights, but the materials will be very useful for teaching.

Response:

We agree with the Editor and reviewer 2, to rearrange the manuscript in the letter format. Thus, we have prepared the edited version of our manuscript accordingly. To fit the new shorter format, the figures of the original manuscript were transferred to the supplemental material. Two new figures were assembled for the main manuscript, which highlights the best responding lines and treatments. The supplemental material includes additional text describing the reproducibility and limitations of the RUBY lines.

Reviewer comments:
Reviewer #1:

In this study, different promoters were selected to transcribe RUBY, such as RAB18, JAZ10, ZAT11 (ROS reporter sensor), promoters WRKY40, WRKY75 (involved in various environmental stress regulation processes), and 35S and UBQ10 (constitutive promoters). These vectors were transferred into Arabidopsis Thaliana by flower infection, and two T3 lines were selected to evaluate the effects of ABA, MeJA, biological stress, and chemical stress on the expression of different Promoter-RUBY transgenic events. During the construction of 35S-RUBY stable coloring Arabidopsis Lines, gene silencing occurred in many lines, which may be caused by epigenetic modification. In addition, 35S-RUBY plants showed hyponastic growth. These were worthy for teaching discussion. I have the following concerns that need to be addressed by the authors.

Major concerns
1. It is better to use UBQ10-RUBY stable line as positive control in some treatment or stress experiment.

Response:

Thank you for the suggestion. UBQ10-RUBY stable line could be used as a positive control in selected experiments. However, as UBQ10 is constitutively expressed throughout the plant growth cycle even without the aid of any treatments, we believe that it does not add more information regarding the treatments or stress conditions used in the experiments.

2. Please use statistical numbers to show the significance of the influence of the treatment or stress on promoter transcription? Such as the percentage of RUBY color plants/total treated plants.

Response:

Thank you for the comment. In the revised version of the manuscript, we have included the percentage of RUBY color plants/total treated plants in new supplemental table S2 (page 6 of the supplemental file). We also discuss the reproducibility of experiments with RUBY lines on page 5 in the supplemental file.

3. The production of genetically modified materials is a general situation for all types of genetic transformation materials and should not be a special problem for RUBY.

Response:

We agree that use of genetically modified materials is not a special problem in the context of making RUBY plants. However, we are affiliated with a University that has full permission to use GMO modified plant materials (in the Finnish system they are GMO class 2). While making the RUBY lines as a teaching resource, we have also been in contact with units at the University of Helsinki that are engaged in public outreach towards high school students, and here we realized that they would currently not be able to grow the plants due to lack of the relevant permission. As we hope that the RUBY lines could be used not only at university level education, we think it is important to point out that their use require compliance with relevant GMO legislation. As we agree that this is not one of the main points in the article, we have moved it to supplemental text.

4. Some other literature has shown that strongly expressed RUBY can affect plant growth. In this experiment, multiple promoters were used to transcribe RUBY, but there was no relevant explanation. Please show and explain the effect of strongly expressed RUBY on plant growth rate and plant size.

Response:

It is true that strong RUBY expression can affect plant growth. Some articles, such as Jogam et al. 2024 (https://link.springer.com/article/10.1007/s11240-024-02752-2) have mentioned that RUBY can affect plant growth. In their experiments, the RUBY-expressed plants exhibited slightly reduced plant height and delayed flowering time in tobacco compared to the wild-type plant. In our lines, the stress inducible promoters (*JAZ10*, *LURP1*, *RAB18*, *WRKY40*, *WRKY75* and *ZAT12*-RUBY) are identical in size to wildtype, see supplemental figures S2-S4.

As can be seen in for example Jogam et al, the question about impact of RUBY on growth, has been addressed when constitutive promoters are used. Here our results are mixed with Arabidopsis 35S- and UBQ10-RUBY lines. In some repeats the plants are smaller, while in other repeats the plants are similar in size to wildtype. We agree with the reviewer that this is an important question to address, but we think this is a topic that require more detailed characterization and would be better explored in a separate study. For example, it would be valuable to also follow photosynthesis – are the purple leaves in 35S and UBQ10-RUBY shielding the leaves from light leading to less efficient photosynthesis? In the previous version of the manuscript we also showed and discussed that 35S-RUBY plants show hyponastic growth, this has been removed from the new version of the manuscript to fit into the shorter letter format.

5. Figure 5 panel b xii is red seeds produced by green plants? Control photos of plants, inflorescences and seeds identical to T0 and T1 of WT are required.

Response:

Yes, to our surprise, the plants of line C2-3-2 of 35S-RUBY were green in color, and the siliques were green, while the seed produced by these green plants were red. As *Arabidopsis thaliana* (Col-0) is a highly exploited and characterized model organism, it is firmly established that the WT plants have green inflorescences, and the seeds are green. While we are aware that it may be a good comparison, owing to space limitations, we did not include the inflorescences of WT plants in our previous or revised version. The inflorescence of Col-0 WT is shown below.

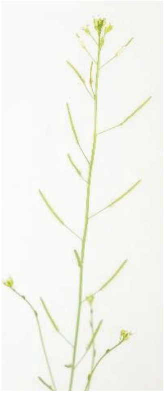

6. In discussion, the author mentioned the UV/Vis-spectra is different for betalain (UV/Vismax at 534 nm) (Chaux-Gutiérrez et al., 2021) and anthocyanin (UV/Vismax at 512 nm) (Young and Abdel-Aal, 2009). Please show some results about this from your experimental plants.

Response:

Thank you for pointing this out. Arabidopsis plants when stressed (either due to age, drought or other unfavorable growth conditions), can produce anthocyanins, which is a pigment clearly visible to our naked eyes and is a marker of plant response to stressed conditions. Though both pigments (anthocyanin and betalains) look partially similar to the naked eye, they are distinct from each other with different UV/Vismax.

To assist students and teachers in observation of anthocyanin versus betalain, we prepared new supplemental figure S8, which shows wildtype plants accumulating anthocyanin versus stress inducible promoter-RUBY lines (in this case exemplified with *WRKY75*-RUBY). Anthocyanins accumulate uniformly across leaves, while RUBY is localized to the area of the treatment used to activate the promoter. As pointed out in our recommendations for using the RUBY lines in teaching (due to the revised manuscript being a shorter format, this is now located at pages 4-5 in supplement), we recommend growing Col-0 side by side with RUBY lines. This makes it possible to see if Col-0 accumulates anthocyanins in the growth conditions or treatments used by teachers and students and to distinguish between anthocyanin versus betalain accumulation.

Minor concerns

1. In Figure 4, the plant styles selected in the photo should be consistent, single plant, two plants, or multiple plants.

Response:

Thank you for your response. We apologize for our inconsistencies with the figures. We initially decided that having one plant to show the treatment effects will offer more meaningful information. Now, in the revised version, as per your suggestions, we have included all plants (4 plants per pot) to make it consistent with the rest of the treatments throughout the figure. This figure is now supplemental figure S4.

Reviewer #2 (Comments for the Author):

RUBY as a reporting system to develop plant materials for teaching purposes is an excellent concept. However, as mentioned in the discussion section, the use of promoter-driven expression of marker genes through different signaling pathways presents a challenge due to longer response times. This is the disadvantage of transcriptional regulatory systems. The manuscript provides only a brief analysis of the phenotype and application specificity of the reporting system, and its scientific and theoretical innovation significance does not meet the scope for a Research Article in Plant Physiology. I recommend considering a submission to Plant Physiology Letters instead. Several issues need to be addressed:

Response:

Thank you for your detailed and kind response. As indicated in our response to the Editor, the new version of the manuscript has been edited to the Letter format.

1. Lines 79-81 are duplicated.
Response:

Thank you for spotting this. The duplication has been removed in the revised version of the manuscript.

2. The photo in Figure 2 is too dark; increasing the overall brightness is recommended.
Response:

Thank you. The brightness of the figure has been modified as suggested (this figure is now Supplemental figure S2).

3. As shown in Figure 3 (XX), the plant has bolted, making it unsuitable for bacterial infection experiments.
Response:

Here we used 3.5 week old plants grown in 12/12 light/dark cycle. At this age the plants have not bolted. Bolting typically occurs at 5-6 weeks under these conditions in our growth chambers.

4. To detect reporter gene expression more rapidly, the authors might consider increasing the concentration of hormones or stress treatments, allowing students to observe the phenotype within class hours.

Response:
Our original plan with RUBY was to also use selected promoter-RUBY lines in research on reactive oxygen species signaling. Here we treat plant with the air pollutant ozone to strongly active signaling. In ozone treatment several thousands of genes are strongly up-regulated within two hours, including *ZAT12*, *WRKY40* and *WRKY75*. To our surprise we only see RUBY color in our *ZAT12*- and *WRKY75*-RUBY lines the day after ozone treatment. Hence, we performed qPCR with primers for the RUBY transcript to see if the failure was in production of the RUBY mRNA or the RUBY color. As can be seen from the qPCR results, the RUBY transcript accumulates to high levels early (2 hr) in the treatment. Despite this, we only see RUBY color in leaves on the next day. Our suggestion is that the synthesis of betalain in sufficient amounts to be seen with naked eye in Arabidopsis leaves takes time – in our hands overnight.

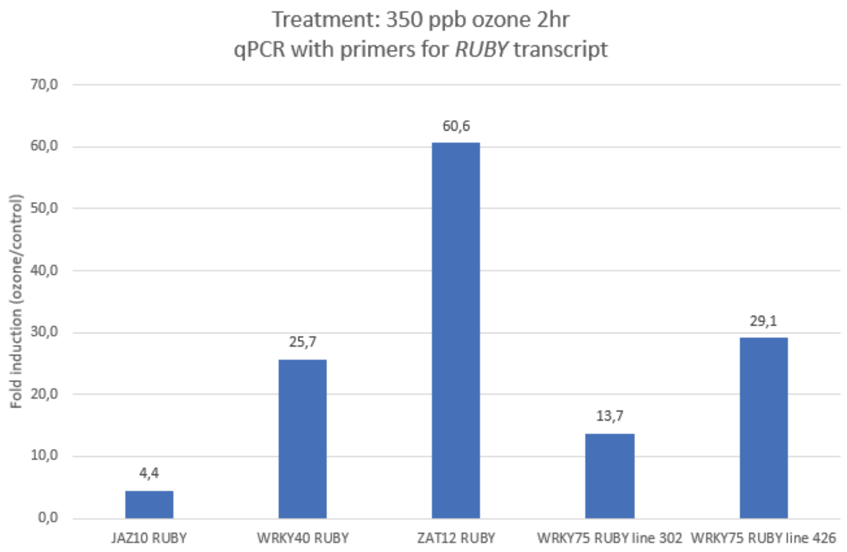

Finally, we would like to thank the editor and reviewers for their insightful and helpful comments on our manuscript. These comments helped us to reformat our manuscript to a new shape. Thank you!

08-Jul-2024
Dr. Mikael Brosche
Helsingin yliopisto
Helsinki 00014
Finland

MSID: PP2024-RA-00643R1
MS TITLE: Seeing the invisible - new tools to teach and study plant transcriptional responses

Dear Dr. Mikael Brosche:

I am pleased to inform you that your revised manuscript "Seeing the invisible - new tools to teach and study plant transcriptional responses" has been accepted for publication in Plant Physiology. Your manuscript will be checked for consistency with journal formatting and image standards by one of our Science Editors, who will contact you shortly. They may have a number of queries and suggestions for you. Please keep in mind that they work to increase the online visibility of your article and its attraction for readers from the widest of backgrounds. You will have the opportunity to review any revisions, and I hope you will work constructively with them to get the most out of your research. You will also have the opportunity to review all changes when you receive the author proofs for your article.

Once validated for publication, your article will be sent to a Science Editor for a final review of your manuscript and source files. After your final files are uploaded, your article will appear in the next available issue and the online Preview version of your article will be posted after your signed Copyright and Checklist forms and final source files are received.

Please be sure to include the following in your manuscript file:
Harvard style citations
List of all author contributions and funding information at the beginning of manuscript after the titles and author list.
List all supplemental materials associated with the article, with titles, at the end of the ms, before the references and acknowledgments section.
Complete Figure Legends for all figures appearing in the article listed before the Literature Cited.

Thank you and congratulations on your Plant Physiology paper!

Sincerely,
Plant Physiology

=====
FIRST AUTHOR PROFILES
=====

We recognize the hard work that goes into being First Author of a Plant Physiology paper by publishing a "First Author Feature" for each First Author alongside your paper.

If the first author(s) would like to submit information for a feature on Facebook and Twitter, fill out the form by clicking on the link below:

Plant Physiology First Author Feature Form: https://forms.office.com/r/ZBCGZfhm8F
If you cannot access the form through the link above, please contact Rachel at rbelsky@aspb.org.

To upload an image of yourself to accompany your profile, please put your photo in the correct journal folder at the link below. You must label your image: Author First Name Last Name Article Title.

First Author Profile Image Dropbox: https://www.dropbox.com/scl/fo/xz029p7qjr2jml2rp2kmq/h?dl=0&rlkey=6fkxcaqmxx35ap152b2osy1fz

=====
IMPORTANT REMINDER: PEER REVIEW REPORTS

=====

If you opted to publish a peer review report along with your article during the original submission process, it will be prepared by the editorial staff and publicly posted with your manuscript, inside the zip file that contains any other supplemental material. As a reminder, the peer review report is a public record of all comments from editors and reviewers, as well as your prior responses, as you received them in the decision letters for each draft of your manuscript. If you agreed to publish this report and have changed your mind, or are not sure if you selected this option, please contact the editorial office as soon as possible before signing the license agreement from our publisher.

-- Reviewer comments:
Reviewer #1 (Comments for the Author):

The authors have addressed all my concerns and questions. I recommend the manuscript be accepted for publication.

Reviewer #2 (Comments for the Author):

Thank you for your revision.
